# Supplementary material for: Antifungal Activity of Antimicrobial Peptides and Proteins against Aspergillus fumigatus
Source: J Fungi (Basel). 2020 May 18;6(2):65. doi: 10.3390/jof6020065 (PMC7345740; doi:10.3390/jof6020065)
Supplement: Supplementary file 1 [file jof-06-00065-s001.pdf]

Supplementary Materials

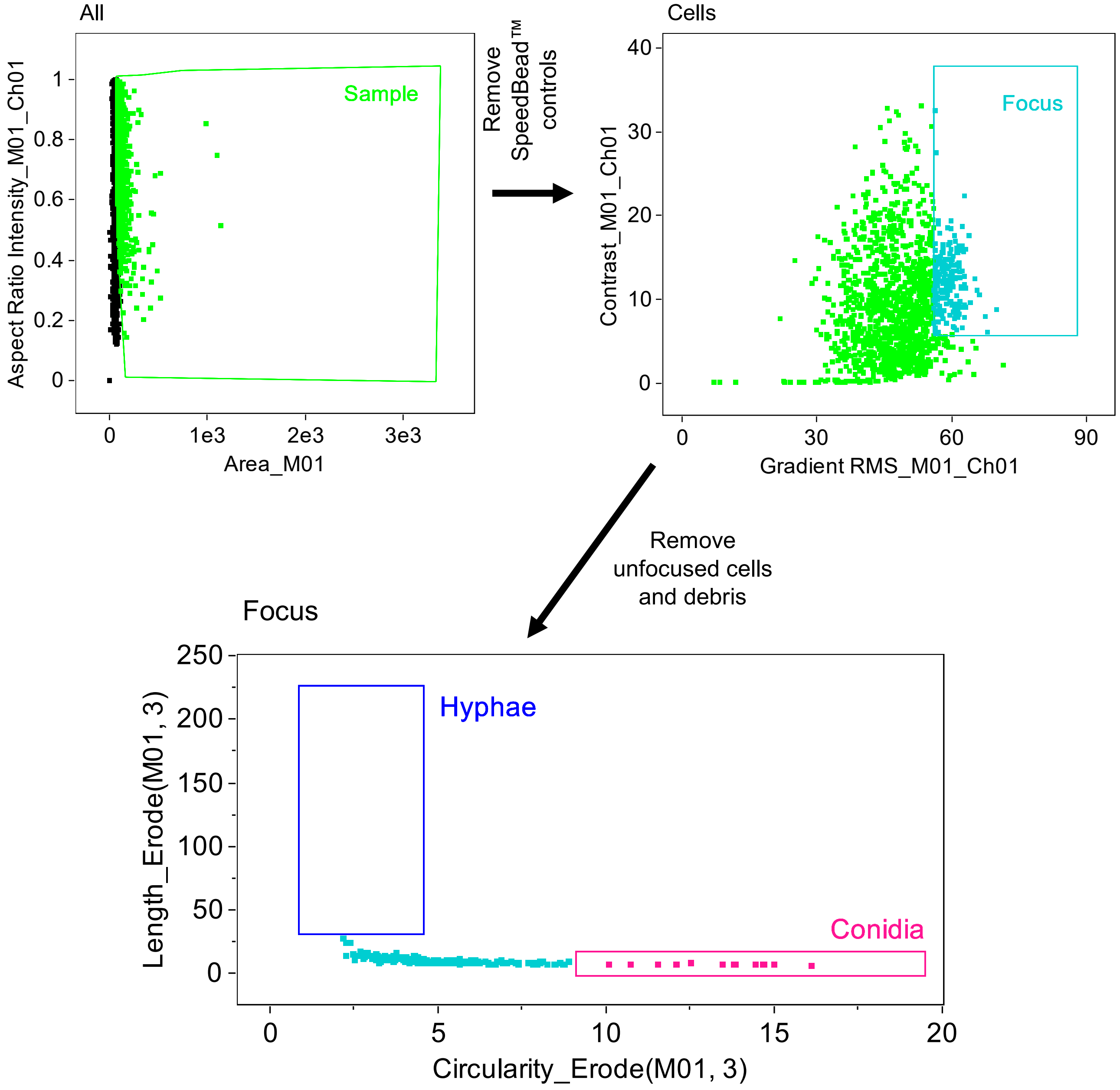

Figure S1. Data cleaning strategy for ImageStream data. Data shown is from the 0 h timepoint of the initial characterisation: conidia, pink box; germlings, cyan box; hyphae, dark blue box. Initial screening based on aspect ratio intensity and area was performed to remove Speedbead^{TM} controls, followed by contrast and gradient filtering to remove unfocused cells and debris. Final gating using length vs circularity features based on analysis of images was selected.

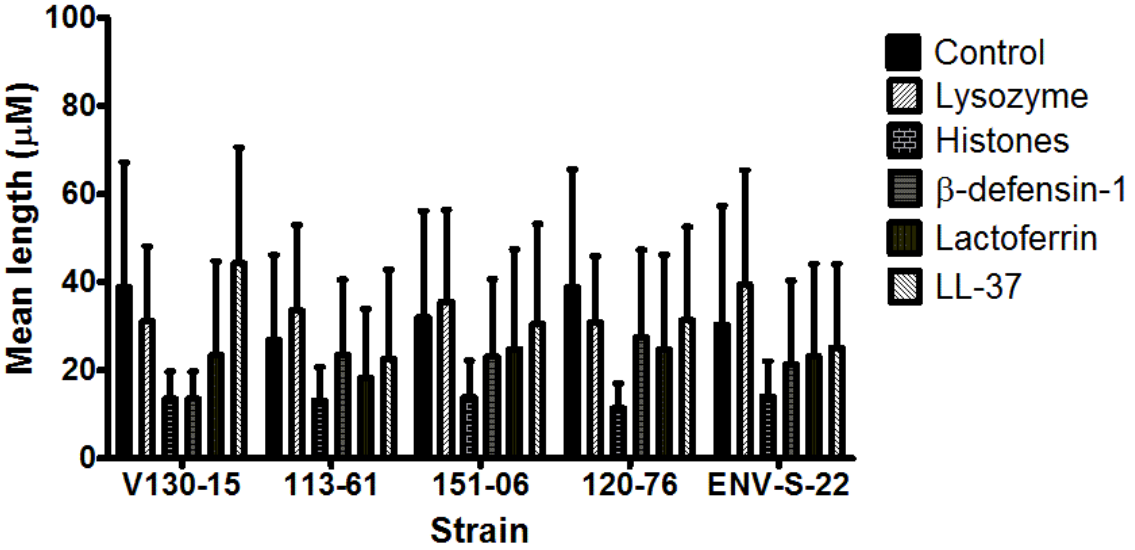

Figure S2. Mean lengths of cells in the hyphae gate under each condition. Strains were incubated with antimicrobial peptides for 10 h at 37°C. Antimicrobial peptide concentrations were: 80 μM lysozyme, 100 μg/ml histones, 10 μM β-defensin-1, 40 μM lactoferrin and 12.5 μM LL-37. After incubation, cells were fixed in 4% paraformaldehyde and analysed using imaging flow cytometry. Data shown is the mean value of all cells in the hyphae gate. Date represents a single experiment per strain and mean values strains ± SD are shown.

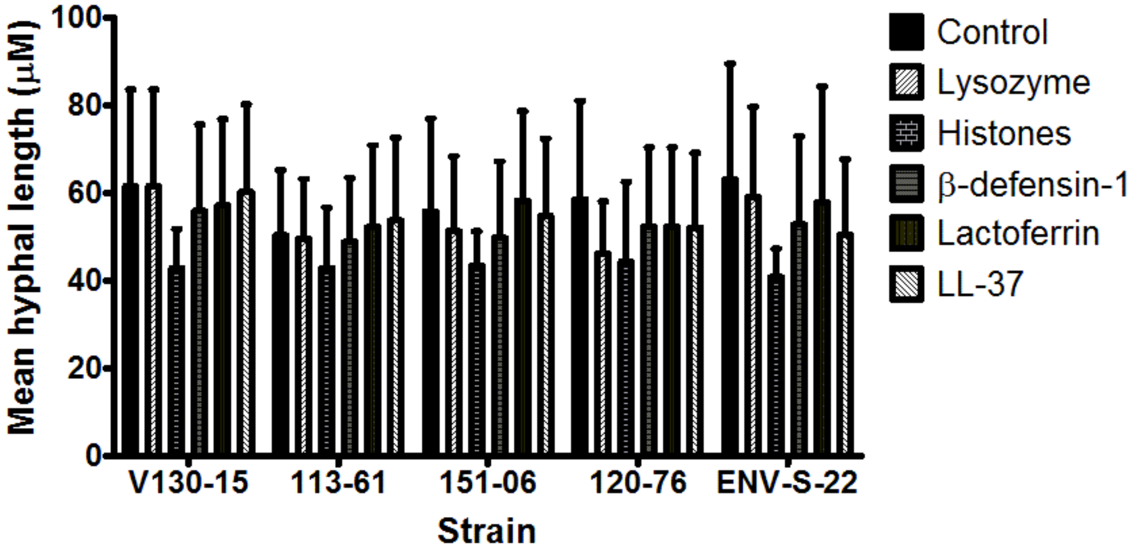

Figure S3. Overall length of all fungal cells under each condition. Antimicrobial peptide concentrations were: 80 µM lysozyme, 100 µg/ml histones, 10 µM β-defensin-1, 40 µM lactoferrin and 12.5 µM LL-37. After incubation, cells were fixed in 4% paraformaldehyde and analysed using imaging flow cytometry. Data represents a single experiment per strain and mean values of all cells measured ± SD are shown.
